# Feasibility of Infrarenal Abdominal Aorta Balloon Occlusion in Pernicious Placenta Previa Coexisting with Placenta Accrete

**DOI:** 10.1155/2018/4596189

**Published:** 2018-06-06

**Authors:** Na Li, Tian Yang, Caixia Liu, Chong Qiao

**Affiliations:** ^1^Department of Obstetrics and Gynecology, Shengjing Hospital of China Medical University, Shenyang 110004, China; ^2^Key Laboratory of Maternal-Fetal Medicine of Liaoning Province and Key Laboratory of Obstetrics and Gynecology of Higher Education of Liaoning Province, Benxi 117000, China

## Abstract

**Objective:**

To evaluate the efficacy and safety of prophylactic balloon occlusion of the infrarenal abdominal aorta in pernicious placenta previa coexisting with placenta accrete.

**Methods:**

This retrospective study was performed in patients with placenta accreta complicated with pernicious placenta previa between January 2014 and December 2016 in Shengjing Hospital; 56 patients with a pathological diagnosis were included. The degree of placental invasion was evaluated by preoperative color Doppler ultrasonography and/or magnetic resonance imaging, and all patients in this study should undergo balloon occlusion preoperatively, which was a determination made by specific doctors. The control group consisted of 32 patients who underwent cesarean section alone, and the study group included 24 patients who underwent cesarean section with preoperative balloon occlusion. Prevention of hysterectomy was the primary outcome evaluated. The secondary outcomes include operative duration, estimated blood loss, blood transfusion, prothrombin time postoperatively, decrease in the hemoglobin level postoperatively, intensive care unit admission, pathological diagnosis, and total hospital stay (days), and these data were compared between the two groups. Additionally, the neonatal outcomes, premature delivery, Apgar scores at 1 minute and 5 minutes, neonatal birth weight, hospitalization, and mortality were compared.

**Results:**

There was a significant difference in the rate of hysterectomy between the two groups (*p*<0.05). However, no differences were observed between the groups in any other outcomes.

**Conclusion:**

The prophylactic use of infrarenal abdominal aortic balloon occlusion is an effective and safe option for treating pernicious placenta previa coexisting with placenta accreta.

## 1. Introduction

Placenta previa refers to the condition where the placenta partially or completely obstructs the internal orifice of the cervix by lying in the lower uterine segment [1]. It occurs in approximately 4.8 per 1,000 pregnancies [2] and might result in maternal mortality and increasing number of maternal morbidities, including massive hemorrhage, infection, adjacent organ damage, and emergency hysterectomy [3, 4]. In past decades, women increasingly preferred to deliver by cesarean section (CS), leading to the increasing incidence of pernicious placenta previa [5]. Pernicious placenta previa, which is the condition when the placenta attaches to previous cesarean delivery scars [6], is often related to placenta accreta. Sumigama et al. [7] reported that the percentage of women who previously delivered by CS and were complicated with placenta increta or percreta was 37% in all cases of placenta previa.

During the operative treatment of pernicious placenta previa, reducing blood loss is a challenge. In 1992, Bakri introduced intrauterine balloon tamponade for treating the obstetric hemorrhage during cesarean delivery [8]. Numerous studies have shown that balloon tamponade successfully controls hemorrhage from the lower uterine segment due to placenta previa accreta. Nowadays, a growing number of obstetricians have effectively applied it to manage intraoperative hemorrhage in patients with placenta accrete. However, intrauterine balloon tamponade may increase CS scar dehiscence [9], uterine rupture, and infection [10] after CS, and when it is used with compression sutures, it may lead to uterine necrosis [11]. Moreover, Kong et al. [12] thought placenta accreta was the adverse prognostic factor associated with higher failure rates when intrauterine balloon tamponade was used in the management of placenta previa.

In previous decades with the development of medicine and technologies, the arterial occlusion balloon was introduced and used in pernicious placenta previa. Iwata et al. suggested that blocking blood flow in the internal iliac arteries was not enough because blood flow from the external iliac arteries was also observed in placenta previa accrete [13]. Further, the efficacy of prophylactic balloon occlusion of the infrarenal abdominal aorta is unclear because of the limited amount of literatures and cases. Therefore, our study aimed to evaluate the clinical efficacy of prophylactic balloon occlusion of the infrarenal abdominal aorta in patients with pernicious placenta previa coexisting with placenta accreta.

## 2. Materials and Methods

### 2.1. Patients

We performed a retrospective study of patients with pernicious placenta previa coexisting with placenta accreta at Shengjing Hospital, China Medical University, Shenyang, Liaoning between January 2014 and December 2016. The inclusion criteria were placenta previa diagnosed by color Doppler ultrasonography or magnetic resonance imaging (MRI), a history of at least one previous CS, and patients without other obstetric diseases. The exclusion criteria were patients with missing clinical data and those complicated with other obstetric diseases.

Finally, 56 patients were included in this study. The degree of placental invasion in every case was evaluated by preoperative color Doppler ultrasonography and/or MRI, and the decision to perform prophylactic balloon occlusion was made by specific doctors. The doctors fully informed women of the benefits and complications of prophylactic balloon occlusion of infrarenal abdominal aorta, and then the women chose to participate in either the balloon group (n=24) or control groups (n=32) based on their conditions. All the women provided informed consent.

Patients' clinical data were evaluated. All women underwent an ultrasonographic examination of the vasculature of their lower limbs at 3 and 42 days postoperatively.

This study was approved by the ethical committee of Shengjing Hospital, China Medical University and supported by the National Key Research and Development Program of Reproductive Health & Major Birth Defects Control and Prevention in China.

### 2.2. Treatment Methods

In the control group, women underwent CS to terminate the pregnancy. After delivery, uterine artery ligation or uterine cavity filing with ribbon gauze or Bakri balloon tamponade was performed when hemorrhage was not stopped. If hemorrhage was beyond control, hysterectomy was performed.

In the study group, women underwent CS and were treated with balloon occlusion of infrarenal abdominal aorta by an experienced interventional radiologist. After subcutaneous local anesthesia, the right femoral artery was punctured using the Seldinger technique, and a 5-French (F) sheath was inserted. Under the guide of wire, we inserted a 5-F catheter into the abdominal aorta along the sheath and performed angiography to visualize blood flow of the abdominal aorta, the renal artery opening, and position of the common iliac artery bifurcation. An 8-F sheath was inserted after withdrawing the 5-F catheter, and the Bard 16-40-mm balloon catheter was inserted into the abdominal aorta at the level of the renal artery along the 8-F sheath. The sheath/balloon catheter system was affixed to the skin. Subsequently, the patients were transferred from the interventional radiology suite to the operating room and underwent CS after general anesthesia. Immediately after delivery, the balloons were inflated using a predetermined volume of normal saline with pressure below 6 atmospheres. The duration of occlusion was recorded for all patients. The longest single continuous occlusion could not last more than 60 minutes, and deflating the balloon for about 10 minutes should be performed when 45 minutes of continuous aortic occlusion occurs. The balloons were routinely deflated before closing the peritoneal cavity to confirm hemostasis. When massive hemorrhage occurred, the balloons were deflated, and the following procedures were performed: uterine artery ligation, uterine cavity filling with ribbon gauze, or Bakri balloon tamponade. Hysterectomy was performed if the hemorrhage was beyond control. The catheters were removed postoperatively, and patients' vital signs were closely monitored.

In all cases, pathological examination of the manually removed placenta attached to the uterus was performed.

### 2.3. Outcomes

The primary outcome was prevention of hysterectomy.

The secondary outcomes were duration of the operation, estimated blood loss, blood transfusion volume, prothrombin time (PT) postoperatively, decrease in the hemoglobin (HGB) level postoperatively, intensive care unit (ICU) admission, pathological diagnosis, and total hospital days. Neonatal outcomes, premature delivery, Apgar scores at 1 minute and 5 minutes, neonatal birth weight, hospitalization, and mortality were also evaluated.

### 2.4. Statistical Analysis

The clinical data were analyzed by the SPSS 22.0 software (IBM Corp.). The categorical data are expressed as a number or proportion and analyzed using the *χ*^2^ test or Fisher exact test. The continuous variables are expressed as the mean ±standard deviation (SD) and analyzed by two-sample t-test and Mann–Whitney U test if the data were not normally distributed. A* p* value < 0.05 was considered statistically significant. The statistical power was evaluated by the PASS 11.0 software (PASS 11.0 software).

## 3. Results

Fifty-six women who were diagnosed as having pernicious placenta previa (low-lying, 2; previa partialis and marginalis, 6; and previa totalis, 48) met the inclusion criteria and underwent CS. There were 24 cases in the study group and 32 in the control group.

Maternal demographic and clinical characteristics between the two groups are shown in Table 1. The two groups did not significantly differ in terms of their maternal age, gravity, parity, or medical history, such as previous vaginal or cesarean delivery, and history of myomectomy or other gynecologic surgery. There was no difference in the percentage of placenta positions and types of placenta previa between the two groups.

There were significantly more patients with hysterectomy in the control group than in the study group (50.0% versus 8.3%,* p*=0.001), with 93% power. No significant differences were observed between the groups in the duration of surgery, estimated blood loss, blood transfusion volume (including packed red blood cell [PRBC] transfusion and fresh frozen plasma [FFP] transfusion), PT postoperatively, decrease in HGB level postoperatively, ICU admission, pathological diagnosis, and total hospital stay (Table 2). Although there was no significant difference in the need for blood transfusion or the mean amount of blood products transfused, differences in the volumes of PRBCs and FFP transfused were 1.5 U and 200 ml, respectively, between the study group and control group (5.83 U versus 7.39 U; 546 ml versus 740 ml). Moreover, the mean estimated blood loss was lower in the study group than in the control group (1600 ml versus 2032 ml). Regarding massive hemorrhage, 7 and 10 patients underwent uterine artery ligation in the study and control groups, respectively (29.2% versus 31.3%,* p*=0.867).

There was no significant difference in the pathological diagnosis between the study and control groups. The mean time for balloon inflation was no more than 25 minutes (23.81 minutes; range, 5-50 minutes). There was a higher percentage of ICU admissions in the study group than in the control group (37.5% versus 28.1%,* p*=0.457), but this was not a significant difference likely because of the precautions followed to prevent severe complications. There was no maternal mortality in either group; all the mothers were healthy at discharge. One woman in the study group developed thrombosis of the right external iliac artery and recovered after operative treatment.

There were 24 live births in the study group and 30 in the control group, excluding two neonatal deaths (one neonate died preterm at 26 weeks' gestation and the other died of congenital malformation). The neonatal characteristics are listed in Table 3. There were no differences in premature delivery, Apgar scores at 1 minute and 5 minutes, the neonatal birth weight, hospitalization, and mortality between the two groups, and there was no recorded neonatal Apgar score less than 7 at 5 minutes in both groups.

## 4. Discussion

In this study, we found a lower percentage of hysterectomies in the study group than in the control group and no other significant differences between these groups.

Chen et al. [14] and Wu et al. [15] reported that prophylactic use of aortic balloon occlusion can reduce the need for hysterectomy. However, they also suggested that balloon occlusion of lower abdominal aorta was effective in reducing intraoperative blood loss and blood transfusion, which were not significantly different in our study groups. In a retrospective study, Xie et al. [16] found that percentage of women with placenta accreta who required cesarean hysterectomy was not significantly different between the balloon group and control group. Additionally, Cui et al. [17] reported that the operative time, blood transfusion volume, change in HGB level, hysterectomy, and length of hospitalization did not differ between the balloon group and the nonballoon group. The clinical efficacy of aortic balloon occlusion has been described by various authors with different results, which might result from the different inclusion and diagnostic criteria for placenta accreta in different studies. In this study, all the women had a history of at least one previous CS and were diagnosed as having pernicious placenta previa, which was usually associated with placenta accreta and led to massive intraoperative massive hemorrhage. In addition, evaluation of the degree of placenta invasion was made by using preoperative color Doppler ultrasonography and/or MRI, and the diagnosis was validated by pathological finding. However, an intraoperative diagnosis was made in most studies.

Pathological examination should be performed because the intraoperative diagnosis is limited; thus, it may not be very accurate to determine the degree of placental invasion, and surgeon experience could affect its accuracy. In the present study, after CS, the manually removed placenta attached to the uterus was sent to the Department of Pathology in order to further verify and determine placental invasion and placenta accreta. The pathological diagnosis is a postoperative diagnosis, which is a medical step far from meaningful treatment. Eshkoli et al. [18] found that a pregnancy following a previous occurrence of placenta accreta is at an increased risk for adverse maternal outcomes. In a literature review, Cunningham et al. [19] reported that the recurrence risk of placenta accreta in subsequent pregnancy following conservative management of placenta accreta is 20%. The pathological diagnosis was crucial in guiding the course of subsequent pregnancies, preventing adverse maternal outcomes, and improving perinatal outcomes. Preoperational examination of the degree of placental invasion was helpful in choosing different methods for preventing hemorrhage. Currently, methods are increasingly used to prevent hemorrhage in patients with placenta previa and placenta accreta during cesarean delivery, such as improvement of suturing technology, intrauterine balloon tamponade, interventional arterial or internal iliac artery ligation, interventional arterial radioembolization, balloon occlusion of the artery, and hysterectomy [13, 20, 21]. However, all these methods have limitations, and their clinical efficacy is uncertain because of technical difficulties, increased duration, and the requirement for complex equipment [22]. In our center, we introduced preoperative prophylactic placement of a balloon catheter for occlusion of the infrarenal abdominal aorta to reduce hemorrhage due to pernicious placenta previa coexisting with placenta accreta during the operation. As a new technique, it can block the uterine blood supply tentatively and completely with no influence from the arterial branch. In addition, it can provide a relatively clear surgical field while exposing the hemorrhage site in order to save time during the operation and provide the opportunity to preserve the uterus. Careful attention is needed to ensure that it does not affect the blood supply to the ovaries and kidneys [23]. The current study showed that the study group resulted in less blood loss and fewer blood transfusion or less mean volume of blood products transfused than the control group despite no significant difference. The average estimated blood loss in the study group was 1600 ml (SD1185.875 mL), and this is nearly the same as that in Clausen et al.'s report [24], in which the average estimated blood loss was 1650 ml (SD 1100 ml) in the patients in whom endovascular balloon occlusion of the common iliac arteries or aorta was performed.

In this study, we found the rate of uterine artery ligation was higher in the study group than in the control group, although there was no significant difference. The placenta attached to the previous CS scar with higher incidence of placenta accreta in pernicious placenta previa. When the placenta was stripped, massive refractory bleeding occurs due to myometrial loss and poor contraction. Once the balloon is deflated, which does not fundamentally solve the problem of postpartum hemorrhage, pelvic blood supply is restored, and other methods should be used, such as uterine artery ligation.

The complications that may occur after infrarenal abdominal aortic balloon occlusion include the following: embolization of the lower extremities, injury of the vascular endothelium, acute renal function injury caused by the change of balloon's position, and vascular rupture. However, the risk of these complications can be reduced or avoided by selecting the appropriate balloon diameter, accurately locating and fixing external parts of the balloon catheter, monitoring renal blood flow, and limiting the occlusion time as much as possible. The occlusion time of the abdominal aorta should not be too long, and it is recommended clinically that the occlusion time should be no more than 60 minutes each time [25]. If a continuous and long-term occlusion is needed intraoperatively, the occlusion should be stopped intermittently for 10-15 minutes to restore the blood supply [26]. Otherwise, some complications associated with balloon occlusion of infrarenal abdominal aorta, such as distal ischemia, reperfusion injury, thrombosis, and embolization of the lower extremities, may occur. The most serious complication is acute renal failure due to the displacement of the balloon catheter and occlusion of the renal artery. In the present study, the mean time for balloon inflated each time was no more than 25 minutes, and there was no intervention-related complication because of our experienced doctors and the strict indications. In our study, occlusion was performed twice in two patients because of massive hemorrhage. We also started the occlusion after delivery; thus, no harm was caused to the newborns. One patient in the study group developed right external iliac artery thrombosis 4 weeks postoperatively; she recovered from the pain and paralysis of the right lower limb after operative treatment (right femoral artery incision and thrombectomy, right external iliac artery balloon dilatation, and stent implantation), returned to normal activities, and had no obvious thrombosis after 1 year postoperatively. Wu et al. [15] reported two patients with vein thrombosis of lower limbs. Thus, ultrasonographic examination of the lower limb vessels is necessary postoperatively. Although infrarenal abdominal aortic balloon occlusion is effective, it does not mean that all the patients with pernicious placenta previa should receive this treatment. By only using this method in the patients who require it to reduce the risk of massive hemorrhage, its misuse can be avoided; however, this is a good way to reduce complications.

Although the abdominal aortic balloon occlusion is widely used in different disciplines as a novel medical technique, it is not widely used in obstetrics. It can reduce intraoperative blood loss, reduce blood transfusion, and significantly reduce the hysterectomy rate, leading to preservation of fertility. Furthermore, there are no such relatively large cases of pernicious placenta previa with pathological diagnosis in previous studies. The clinical data in our study were from Shengjing Hospital, China Medical University, which is a referral medical center in northeastern China that represents the epidemiological trend of the northeastern province of China.

Despite this study's strengths, its limitations should be highlighted. First, the study was limited by a small sample size because of the rarity of this condition. Although the incidence of pernicious placenta previa coexisting with placenta accreta has increased, the total number of cases remains low so only 56 patients were included in this study. Second, the decision to perform infrarenal abdominal aortic balloon occlusion was made by specific doctors; therefore, there was selective bias and the doctors' experience could affect the decision. Third, this study lacked a long-term follow-up because of its retrospective design. In the future, larger investigations involving multiple centers and large numbers of patients should be performed with longer follow-up periods to provide accurate assessment and validation of the clinical efficacy of this method. Lastly, we just compared the prophylactic use of balloon occlusion of the infrarenal abdominal aorta with a control group and did not compare this method with other treatments for avoiding hemorrhage.

In conclusion, prophylactic use of balloon occlusion of infrarenal abdominal aorta is an effective and safe option for the treating pernicious placenta previa coexisting with placenta accrete, as it does not cause harm to the newborn. Additionally, it can prevent women from having to undergo hysterectomy and preserve their fertility.

## Figures and Tables

**Table 1:** Comparison of maternal demographic and clinical characteristics between the study group and control group.

		Study group (n=24)	Control group (n=32)	*p *value
Age (years)		33.75±5.144	33.34±3.442	0.740
Gravity		4 (2-6)	3 (2-7)	0.099
Parity		1 (1-3)	1 (0-2)	0.733
Number of Vaginal deliveries		0 (0-1)	0 (0-1)	0.837
Number of Cesarean deliveries		1 (1-2)	1 (0-2)	0.829
Myomectomy		0 (0.0%)	1 (3.1%)	1.00
Other gynecologic surgery		3 (12.5%)	4 (12.5%)	1.00
Placenta position	Anterior	12 (50.0%)	14 (43.8%)	0.057
	Posterior	1 (4.2%)	9 (28.1%)	
	Anterior and posterior	11 (45.8%)	9 (28.1%)	
Type of placenta previa	Low-lying	0 (0.0%)	2 (6.3%)	0.693
	Previa partialis and marginalis	3 (12.5%)	3 (9.4%)	
	Previa totalis	21 (87.5%)	27 (84.4%)	

Values are presented as the mean ± standard deviation, median (range), and number (percentage).

**Table 2:** Comparison of surgical outcomes between the study group and control group.

		Study group (n=24)	Control group (n=32)	*p *value
Hysterectomy		2 (8.3%)	16 (50.0%)	0.001
Duration of the operation		103.08±56.555	107.38±64.015	0.987
Estimated blood loss (ml)		1600.00±1185.785	2032.81±1881.258	0.727
Transfusion		19 (79.2%)	20 (62.5%)	0.179
	PRBCs (U)	5.83±3.754	7.39±3.794	0.184
	FFP (ml)	546.43±274.888	740.63±428.649	0.064
PT level postoperatively		11.93±1.243	11.70±0.984	0.442
Decrease in the HGB level postoperatively (g/l)		21.17±18.339	22.47±18.263	0.793
ICU admission		9 (37.5%)	9 (28.1%)	0.457
Pathological diagnosis	No	1 (4.2%)	5 (15.6%)	0.335
	Placenta accreta	6 (25%)	6 (18.8%)	
	Placenta increta	17 (70.8%)	21 (65.6%)	
Total hospital stay (days)		8.08±3.810	9.25±5.388	0.390
Uterine artery ligation		7 (29.2%)	10 (31.3%)	0.867
Thrombosis of the lower limbs		1 (4.2%)	0	0.429

Values are presented as the mean ± standard deviation and number (percentage). PT: prothrombin time; HGB: hemoglobin; PRBC: packed red blood cells; FFP: fresh frozen plasma; ICU: intensive care unit.

**Table 3:** Comparison of neonatal outcomes between the study group and control group.

	Study group (n=24)	Control group (n=32)	*p *value
Neonatal birth weight	2928.75±411.776	2698.03±652.810	0.136
Apgar score in 1 minute	8.39±1.644	8.47±1.852	0.754
Apgar score in 5 minutes	9.48±0.790	9.40±0.932	0.983
GA at delivery (weeks)	35.83±1.308	35.22±3.024	0.818
Pre-term birth	16 (66.7%)	19 (59.4%)	0.577
Neonatal hospitalization	6 (25%)	14 (43.8%)	0.147
Neonatal mortality	0 (0.0%)	2 (6.3%)	0.501

Values are presented as the mean ± standard deviation and number (percentage). GA: gestational age.

## Data Availability

The data used to support the findings of this study are available from the corresponding author upon request.
